# Effects of salt and stress on blood pressure parameters and antioxidant enzyme function in the heart and aorta of borderline hypertensive rats

**DOI:** 10.1113/EP090714

**Published:** 2023-05-02

**Authors:** Bojana Savić, Jelena Brkljačić, Sofija Glumac, Olivera Šarenac, David Murphy, Duško Blagojević, Nina Japundžić‐Žigon, Zorana Oreščanin Dušić

**Affiliations:** ^1^ Institute of Pharmacology, Clinical Pharmacology and Toxicology, Faculty of Medicine University of Belgrade Belgrade Serbia; ^2^ Department of Biochemistry, Institute for Biological Research ‘Siniša Stanković’, National Institute of Republic of Serbia University of Belgrade Belgrade Serbia; ^3^ Institute of Pathology, School of Medicine University of Belgrade Belgrade Serbia; ^4^ Bristol Medical School: Translational Health Sciences, Dorothy Hodgkin Building University of Bristol Bristol UK; ^5^ Department of Physiology, Institute for Biological Research ‘Siniša Stanković’, National Institute of Republic of Serbia University of Belgrade Belgrade Serbia

**Keywords:** antioxidant enzymes, borderline hypertensive rats, hypertension, salt, stress

## Abstract

Hypertension and its complications are a leading cause of death in the human population. Several factors can contribute to development of hypertension, such as genetic predisposition, high salt intake and environmental stressors, underlying oxidative stress as one of its key trademarks. We studied the effects of increased salt intake and chronic stress on blood pressure parameters and the activity and protein levels of antioxidant enzymes in the heart and aorta of borderline hypertensive rats (BHRs) with genetic susceptibility to hypertension. All animals were randomized into four groups: (1) Wistar rats kept in baseline conditions; (2) BHRs kept in baseline conditions; (3) BHRs drinking 0.9% saline solution; and (4) BHRs drinking 0.9% saline solution and exposed to repeated heterotypic stress. The BHRs exhibited significantly higher blood pressure, mitochondrial superoxide dismutase (SOD2) and catalase (CAT) protein levels and lower glutathione peroxidase (GPx) and glutathione reductase (GR) activities in the aorta, followed by lower CAT and GPx protein levels and higher CAT and GR activities in the heart, compared with normotensive Wistar rats. In the BHR aorta, high salt intake elevated CAT and GPx activities, and when combined with stress it increased GPx and GR activities. In BHR hearts, high salt intake provoked lower CAT activity. Adding repeated stress to salt treatment further decreased CAT activity, in addition to Cu^2+^–Zn^2+^ superoxide dismutase (SOD1) and GR activities. The protein level of CAT was lower, whereas SOD2 and GPx increased. Overall, our results suggest that BHR hearts are better adapted to oxidative pressure, compared with the aorta, when exposed to salt and stress.

## Abstract (highlights)

- **What is the central question of this study?**
  Although the involvement of reactive oxidative species in triggering hypertension has been documented, there are no data about the role of antioxidant enzymes in the heart and aorta of borderline hypertensive rats kept in baseline conditions or exposed to high salt with or without repeated stress.
- **What is the main finding and its importance?**
  In borderline hypertensive rats, high salt intake and stress contribute significantly to increase blood pressure and antioxidative defence in the aorta but decrease it in the heart. Elucidating the early changes that accompany elevated blood pressure could provide new therapeutical venues for prevention and treatment of the condition.

## 1 INTRODUCTION

Hypertension is a common condition in the human population, and it is projected to affect >1.56 billion people worldwide by the year 2025 (WHO ‐ Hypertension; Elliott, 2006). Thus, it has been recognized as one of the global health priorities for the following period (NCD Risk Factor Collaboration, 2021; Zhou et al., 2021). The modern way of living, characterized by unhealthy dietary habits, including increased salt consumption, sedentary behaviour and frequent exposure to stress, contributes to a high prevalence of hypertension and cardiovascular diseases. Hypertension is considered a leading risk factor for the development of cardiovascular disease and related disabilities, being associated with vascular dysfunction, followed by histological and cellular changes in blood vessels of hypertensive experimental animals and human subjects (Martinez‐Quinones et al., 2018).

Amongst many different possible causes of hypertension, oxidative stress stands out as an important pathological mechanism (Montezano & Touyz, 2012). Oxidative stress is a condition defined by the predominance of pro‐oxidant challenge over antioxidant protection. It is a consequence of either increased reactive oxygen species (ROS) generation or an impaired antioxidant defence system. In excess, ROS induce damage cellular macromolecules, such as proteins, lipids and nucleic acids, thereby affecting cellular function. Nonetheless, ROS, in addition to reactive nitrogen species, play an important physiological role in controlling endothelial function, vascular tone and cardiac function through redox‐sensitive signalling pathways (Valko et al., 2007). Thus, antioxidant defence not only protects tissues, but also regulates the optimal physiological ROS concentration necessary for proper tissue function. Cells use antioxidant enzymes, such as cytoplasmic copper–zinc superoxide dismutase (SOD1), mitochondrial manganese superoxide dismutase (SOD2), catalase (CAT), glutathione peroxidase (GPx) and glutathione reductase (GR), in addition to different non‐enzymatic antioxidant compounds, including reduced glutathione (GSH) and vitamins A, E and C, to regulate the ROS concentration (Valko et al., 2007).

Arterial hypertension is usually studied experimentally in the spontaneously hypertensive rat (SHR) animal model. In these rats, hypertension can be detected at ∼5–6 weeks of age, (some authors describe even earlier onset; Zicha & Kunes, 1999), with blood pressure (BP) reaching maximum values after a week or two. Parallel to BP increase, cardiac output is unchanged with the condition progressing, whereas peripheral resistance is elevated, resulting in blood vessel atrophy. Endothelial impairment happens early, and morphological differences that impact both intimal and media layers are detected from 10 weeks of age, although some authors report vascular changes in fetal and newborn SHRs, attributed to genetically driven factors (Limas et al., 1980). In contrast, human hypertension develops later in life (Williams et al., 2018). Given that SHRs develop hypertension (BP >140 mmHg) before reaching maturity (12 week of age), this makes them an inappropriate model for the study of prehypertensive stages in adulthood. A more adequate genetically predisposed animal model for studying prehypertension in late adulthood is the borderline hypertensive rat (BHR), which is characterized by BP values of ∼140 mmHg. Comparable to the human population, oxidative damage is detected in 22‐week‐old BHRs, implying that changes in redox state could contribute to the development of hypertension (Kluknavsky et al., 2020; Puzserova et al., 2013).

Although the involvement of ROS in the development of hypertension has been documented, data about the role of antioxidant enzymes in BHRs in baseline conditions showed involvement of oxidative stress to some extent, but without reflection on the activity of antioxidative enzymes in erythrocytes (Horvathova et al., 2016), suggesting that oxidative damage progresses slowly. It is well known that additional factors such as crowding stress led to hypertension, but crowding failed to induce oxidative damage to plasma (Bernatova et al., 2018). Data showed that oxidative stress, NO deficiency and endothelial dysfunction are not involved causally in the initiation of BP increase in BHRs, during the peripubertal period (Bernatova et al., 2018). Introducing other factors, such as a high‐salt diet and alternative types of stress, confirmed that the development of essential hypertension in the BHR is not related to oxidative stress and the action of antioxidant enzymes. Therefore, the aim of the present study was to investigate the effects of high salt intake and repeated heterotypic stress on BP parameters and antioxidant enzyme function in the heart and aorta of BHRs, in order to determine how their alterations contribute to the development of hypertension. To that end, BP parameters were measured, morphometric analysis of the thoracic aorta was preformed, and the protein levels and activities of antioxidant enzymes were determined in the heart and aorta of BHRs exposed to high salt intake, with or without chronic stress. In order to validate the animal model and gain a better understanding of basic relationships between oxidative stress and hypertension in BHRs, we estimated differences between untreated normotensive Wistar rats (WRs) and BHRs kept in baseline conditions, before subjecting them to different protocols.

## 2 MATERIALS AND METHODS

### 2.1 Ethical approval

All experiments were performed in compliance with Directive 2010/63/EU of the European Parliament on protection of animals used for scientific purposes and with the UK Animal Act 1986 and ARRIVE guidelines for reporting experiments involving animals (McGrath et al., 2010). The experimental protocol was approved by the Ethics review board, School of Medicine, University of Belgrade (no. 323‐07‐04083/2016‐05/7).

### 2.2 Animals and treatment

In this study, we used male WRs and BHRs. The BHRs were obtained as the first filial generation, by crossbreeding SHR females and normotensive males (Lawler et al., 1988). This strain was previously affirmed in our laboratory by Sarenac et al. (2011), with BP values correlating with normal–elevated to mild hypertension in humans (Mancia et al., 2009).

All animals were matched regarding their age (12 weeks old) and weight (280–330 g) and were housed in a controlled environment (12 h–12 h light–dark cycle, temperature 21 ± 2°C and humidity 65 ± 9% v/v), one animal per cage, with access to standard food pellets (0.2% w/v sodium content; Veterinarski zavod, Subotica, Republic of Serbia) and tap water or 0.9% w/v saline solution ad libitum.

### 2.3 Surgery

Before entering different protocols, all groups of rats underwent surgical procedures under combined ketamine (Ketamidor; catalogue no. A99325611; Richter Pharma AG, Austria; 100 mg/kg, i.m.) and xylazine (Xylased; catalogue no. 1155920A; Bioveta, Czechia; 10 mg/kg, i.m.) general anaesthesia. The depth of anaesthesia was assessed by absence of the corneal and pedal reflexes at the beginning and during the procedure. After shaving the whole abdominal and preparation of the surgical field, the animals were placed on warm temperature pads (Harvard Apparatus, Holliston, MA, USA). A 3‐cm‐long surgical incision was made. The intestines were retracted, and connective tissue was prepared. At the level of renal and iliac arteries, temporary occlusions of abdominal aorta were made in order to slow down the blood flow and facilitate radio‐transmitter catheter placement. The tip of the radiotelemetry device (TA11‐PA C40; DSI, Transoma Medical, St Paul, MN, USA) catheter was guided by a 21‐gauge needle and fixated with a tissue adhesive (3M Vetbond; 3M, USA) and tissue cellulose patch (DSI, Transoma Medical). The body of the radiotelemetry device was sutured to the front abdominal wall, and the incision was closed by classical stiches. Animals were treated perioperatively with gentamicin (catalogue no. J01GBO; Hemofarm, Vršac, RS; 25 mg/kg, i.m.) and carprofen (Rimadyl; catalogue no. 104RI009AE; Phizer, UK; 5 mg/kg, s.c.) to prevent secondary bacterial infection and pain, respectively. After completion of the procedure, rats were placed in transparent Plexiglass cages and monitored until full recovery.

### 2.4 Experimental design

Animals were randomized into four groups and monitored during the 24 week follow‐up period in order to induce overt sustained hypertension. The longevity of the follow‐up was dictated by the technical performance of the radiotelemetry devices. The first group consisted of WRs kept in baseline conditions and drinking tap water for 24 weeks, which were used as normotensive controls for inter‐strain comparison. The second group consisted of BHRs kept in baseline conditions and drinking tap water for 24 weeks (BHR). Third group consisted of BHRs drinking 0.9% w/v saline solution for 24 weeks (BHR‐S). The fourth group consisted of BHRs drinking 0.9% saline solution and exposed to repeated heterotypic stress (BHR‐SS).

In the BHR‐SS group, two blocks of combined stress protocols were alternated for 24 weeks, 6 days a week, followed by a day of rest. The onset of exposure to stress (between 09.00 and 16.00 h) was selected randomly at the beginning of each block, in order to minimize habituation.

The first block lasted 4 weeks and included a combination of shaking and crowding stress protocols, in which animals were exposed to a shaking platform (200 cycles/min) for 30 min daily, while crowding stress was induced by reducing living space to ∼70 cm^2^ per 100 g of animal weight for the entire 4 weeks. The cage size was adjusted using special cages with a flexible wall.

The second block lasted for 6 weeks and involved tilt + isolation + air‐jet stress protocols, in which rats were housed in individual Plexiglas cages (300 cm^2^/100 g) that were tilted (40° incline) for 60 min every day and isolated by a non‐transparent barrier. Additionally, animals were exposed to air‐jet stress for 2 min daily by blowing air on the top of the rat's head.

In all experimental groups, BP was measured in well‐controlled external conditions after animal acclimation, once a week, at the same time, for 30 min (in the stress group, on the first day after the pause in protocol and before the start of the new cycle of stressors). Haemodynamic parameters were assessed after completion of each stress block. The experiments started with nine animals per group, but owing to radio‐transmitter malfunction, not all data were included in the study. The exact number of animals in each group is shown as data points plotted in the figures.

At the end of the experiment, animals were killed by decapitation.

### 2.5 Cardiovascular signal processing and analysis

Signals from the radiotelemetry devices were transmitted to a data acquisition and analysis system composed of a Data Exchange Matrix, PhysioTel Receiver Model RPC‐1 and Ambient Pressure Reference Model APR‐1 (DSI, Transoma Medical) and PC equipped with Dataquest A.R.T. v.4.0 software, (DSI, Transoma Medical) specialized for acquisition and analysis of cardiovascular signals. The arterial pulse pressure (PP) value was digitalized at 1000 Hz and resampled at 20 Hz. Systolic (SBP), diastolic (DBP) and mean (MAP) arterial blood pressures and the pulse interval (PI) or its inverse value, heart rate (HR), were derived from the arterial PP wave as the maximum, minimum and interbeat interval, respectively. The signal length used for analysis was 409.6 s (Savic et al., 2020).

### 2.6 Morphometric measurement

Immediately after decapitation, the thoracic aorta was harvested and split into two sections, used for morphometric and antioxidant enzyme analysis, respectively. One piece of aorta tissue was fixed in paraformaldehyde, dehydrated and embedded in paraffin, sectioned at 5 μm thickness and stained using selective techniques for elastic fibres: the Masson Trichrome technique and staining with the Acid Orcein method (Labudovic Borovic et al., 2010). Images from the dissected specimens were recorded in tiff format with an Olympus DP70 digital camera, and morphometric measurements were performed using a computer‐assisted image analysis system (ImageJ). ImageJ is a public domain, Java‐based image processing program developed at the US National Institutes of Health (Schneider et al., 2012). Histomorphometry was performed by the systematic field sampling method, by examining each section in 10 microscopic fields arranged equidistantly along the circumference of the arterial transection. Only cross‐sections were analysed, and the ocular micrometer had a normal orientation to the vessel wall. The terminal parts of each block, oblique sections, branching points and segments with major technical flaws were excluded from the analysis. The thickness of the intima and the media layers was measured at ×100 magnification, and intima‐to‐media ratio was calculated (Borovic et al., 2013). Using ImageJ, the area delineated by the internal elastic lamina and the lumen area were measured. The stenosis index was computed by subtracting the lumen area from the outer area, and the difference was divided by the outer area. Results were multiplied by 100 to obtain the stenosis index (Nishizawa et al., 2017).

### 2.7 Tissue preparation and collection for antioxidant enyzme analysis

During organ harvesting that followed decapitation, heart was instantly excised along with aorta piece intended for antioxidant enzyme activity and protein level analysis. Both tissues were hear washed in saline, dried and stored in liquid nitrogen at −80°C. There were no remnants of blood and blood clots.

Thawed tissue was homogenized in 10 vol. (w/v) of buffer (50 mM Tris, 0.25 M sucrose and 1 mM EDTA, pH 7.4), sonicated (3 × 15 s at 10 MHz on ice) before centrifugation for 90 min at 105000*g*. The supernatant was used to determine enzyme activities and protein levels.

### 2.8 Determination of antioxidant enzyme activity

Total superoxide dismutase (SOD) activity was determined by the adrenaline method (Misra & Fridovich, 1972). SOD units were defined as the amount of the enzyme necessary to decrease the rate of adrenalin auto‐oxidation by 50%, at pH 10.2. For determination of SOD2 activity, the assay was performed after pre‐incubation with 8 mM KCN. The SOD1 activity was calculated as the difference between total SOD and SOD2 activities. The CAT activity was estimated according to Beutler (1982) by monitoring hydrogen peroxide consumption, expressed in units per milligram of protein. The activity of GPx was determined by the glutathione reduction of *tert*‐butyl hydroperoxide, using a modification of the assay described by Paglia and Valentine (1967). One unit of GPx activity was defined as the amount of the enzyme needed to oxidize 1 μmol of NADPH per minute at 37°C and pH 7. The GR activity was assessed by NADPH oxidation concomitant with glutathione disulphide reduction and expressed in milliunits per milligram of protein. It was determined by the method of Glatzle et al. (1974). All measurements of absorbance were performed using a Shimadzu UV‐160 spectrophotometer (Shimadzu Scientific Instruments, Shimadzu Corporation, Kyoto, Japan).

### 2.9 SDS–PAGE and immunoblotting

After boiling in Laemmli sample buffer, proteins were resolved on 7.5 or 12% SDS–polyacrylamide gels. Western transfer of proteins from gels to polyvinylidene difluoride membranes was performed in 25 mM Tris buffer, pH 8.3 containing 192 mM glycine and 20% (v/v) methanol, at 135 mA overnight in a Mini Trans‐Blot Electrophoretic Transfer Cell (Bio‐Rad Laboratories, Hercules, CA, USA). The membranes were blocked by PBS (1.5 mM KH_2_PO_4_, 6.5 mM Na_2_HPO_4_, 2.7 mM KCl and 0.14 M NaCl, pH 7.2) containing 1% w/v non‐fat milk, incubated by rocking (1.5 h at room temperature) with the primary antibody. The SOD1 (1:2000), SOD2 (1:5000), CAT (1:2000), GPx (1:2000) and GR (1:2000) were detected by Abcam antibodies (ab13498, ab13533, ab16731, ab22604 and ab16801, respectively: Abcam, Cambridge, UK). Endothelial nitric oxide synthase (eNOS) was detected using Anti‐eNOS antibody (1:1000, No: 610296) obtained from BD Biosciences (New Jersey, USA), while β‐Actin (1:5000) was detected by AC‐15 antibody (Sigma Chemicals, St. Louis, MO, USA). After washing with PBS containing 0.1% v/v Tween 20, the membranes were incubated with horseradish peroxidase‐conjugated secondary anti‐mouse or anti‐rabbit IgG antibody (1:30000, ab97046 and ab6721, respectively; Abcam, Cambridge, UK). The immunoreactive proteins were visualized by a chemiluminescence method. Quantitative analysis of immunoreactive bands was done by ImageJ software.

### 2.10 Data presentation and analysis

Statistical analysis was performed according to the protocols described by Wiersma and Jurs (2003). Treatment effect was tested by one‐way ANOVA followed by Tukey's HSD post hoc test. To analyse between‐strain differences, main effect ANOVA was used. Data are presented as means ± SD, and a probability level of *P* < 0.05 was considered statistically significant.

## 3 RESULTS

### 3.1 Effect of salt and stress on systolic, diastolic and mean arterial blood pressures and heart rate in BHRs, and between‐strain differences

**FIGURE 1:**
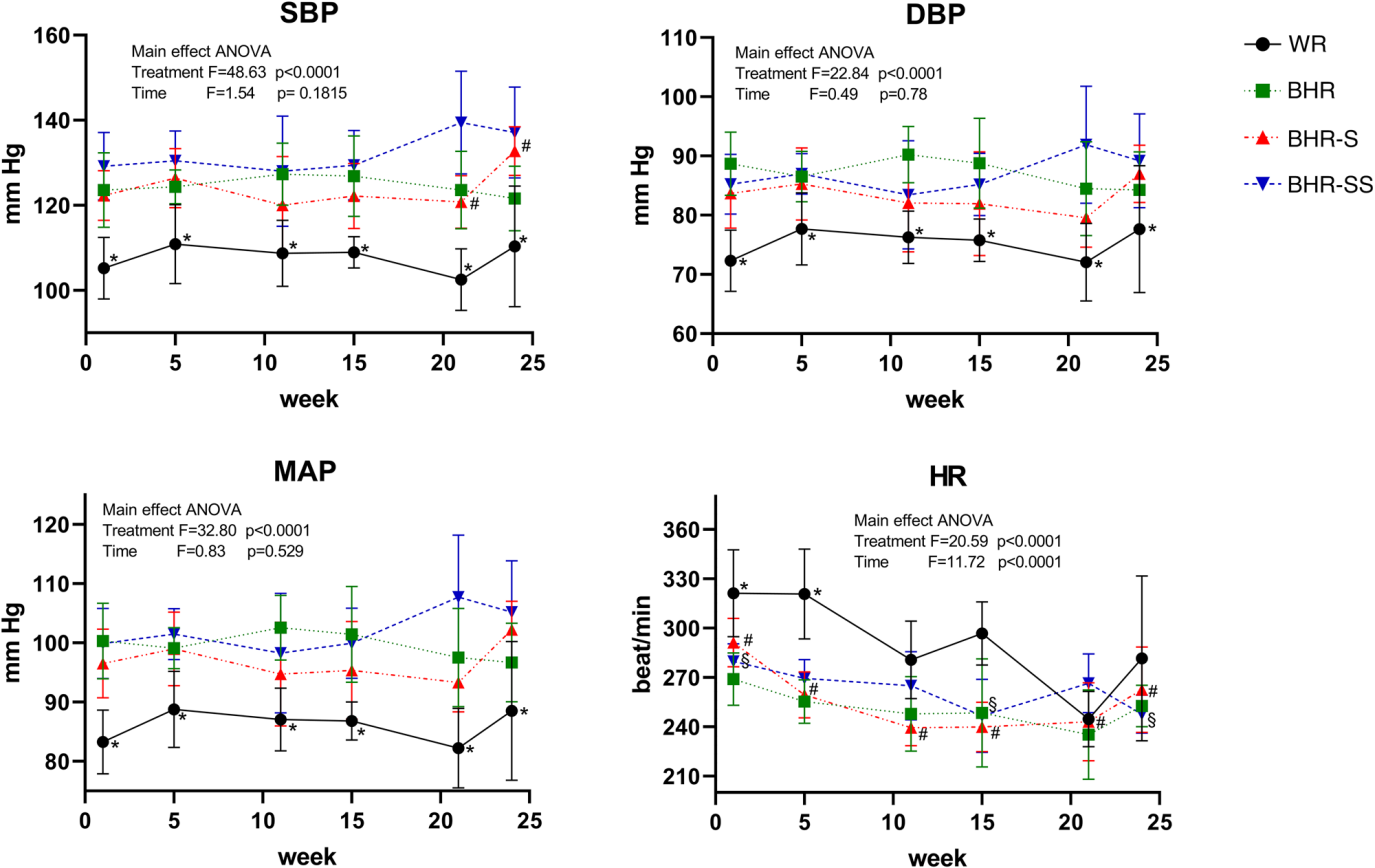
Effect of salt and stress on SBP, DBP, MAP and HR in BHRs, in addition to between‐strain differences. Values are expressed as means ± SD. Statistical significance was tested by main effect ANOVA, with post hoc comparison by Tukey's HSD test. The *F‐* and *P*‐values are presented (^*^statistically significant difference between WR and all BHR groups; ^#^statistically significant difference between time points of BHRs; ^§^statistically significant difference between time points of BHR‐SS). Abbreviations: BHR, borderline hypertensive rat; BHR‐S, borderline hypertensive rat exposed to saline; BHR‐SS, borderline hypertensive rat exposed to saline and repeated heterotypic stress; DBP, diastolic blood pressure; HR, heart rate; MAP, mean arterial blood pressure; SBP, systolic blood pressure; WR, Wistar rat.

The SBP differed between the groups, but there was no effect of SBP time of measurement within individual groups (Figure 1; main effect ANOVA, significant group effect, *F* = 48.63, *P* < 0.0001; no significant time of measurement effect, *P* = 0.1815), except in the BHR‐S group, where elevation of SBP was found in the 24th week compared with both the 11th and 21st weeks. The WRs had lower SBP in comparison to all examined BHR groups (Tukey's HSD post hoc test, *P* < 0.0001). The BHR‐SS group had higher SBP than both the BHR and BHR‐S group (Tukey's HSD post hoc test, *P* = 0.000437 and *P* < 0.0001, respectively).

The DBP also varied between groups, without an effect of time of measurements (Figure 1; main effect ANOVA, significant group effect, *F* = 22.84, *P* < 0.0001; no significant time of measurement effect, *P* = 0.78). The WR group had lower DBP compared with the other groups (Tukey's HSD post hoc test, *P* < 0.0001). However, BHRs in baseline conditions, in addition to the BHR‐SS group, had significantly higher DBP than the BHR‐S group (Tukey's HSD post hoc test, *P* = 0.04397 and *P* = 0.0376, respectively).

The MAP was significantly different between experimental groups, but there was no dissimilarity regarding time (Figure 1 main effect ANOVA, significant group effect, *F* = 32.80, *P* < 0.0001; no significant time of measurement effect, *P* = 0.529). MAP had statistically lower values comparing normotensive WR with other groups (Tukey's HSD post hoc test, *P* < 0.0001); MAP in the BHR‐SS group was significantly higher versus BHR‐S (Tukey's HSD post hoc test, *P* = 0.0025211).

Heart rate was markedly different between measured time points in normotensive WRs (Figure 1; one‐way ANOVA, *P* = 0.003385); HR was significantly higher at both the first week (Tukey's HSD post hoc test, *P* = 0.004771) and fifth week (Tukey's HSD post hoc test, *P* = 0.005035) compared with the 21st week. Heart rate differed statistically between measured time points in the BHR‐S group (one‐way ANOVA, *P* < 0.0001). Heart rate measured at the start was higher compared with subsequent weeks: fifth (Tukey's HSD post hoc test, *P* = 0.01368), 11th (Tukey's HSD post hoc test, *P* = 0.00015), 15th (Tukey's HSD post hoc test, *P* = 0.000156), 21st (Tukey's HSD post hoc test, *P* = 0.00019) and 24th week (Tukey's HSD post hoc test, *P* = 0.034593). Heart rate was also notably different between measured time points in the BHR‐SS group (one‐way ANOVA, *P* = 0.000385); HR was significantly higher in the first week compared with both the 15th and 24th weeks (Tukey's post hoc HSD test, *P* = 0.001289 and *P* = 0.001923, respectively). Main effect ANOVA showed significance in HR regarding both treatments and time (treatment *P* < 0.0001; time *P* < 0.0001). Tukey's HSD post hoc test after the main treatment effect analysed by ANOVA showed that HR was statistically higher in normotensive WRs than in the other groups (*P* < 0.0001 for all comparisons). The same post hoc test after the main time effect analysed by ANOVA showed that HR in the first week was increased compared with the 11th, 15th, 21st and 24th week (*P* < 0.0001 for all comparisons); HR in the fifth week was elevated in comparison to the 15th (*P* = 0.0261130) and 21st week (*P* = 0.0011367).

### 3.2 Effect of salt and stress on morphology of BHR arteries: Intima thickness, media thickness, intima‐to‐media ratio and lumen stenosis, and between‐strain differences

**FIGURE 2:**
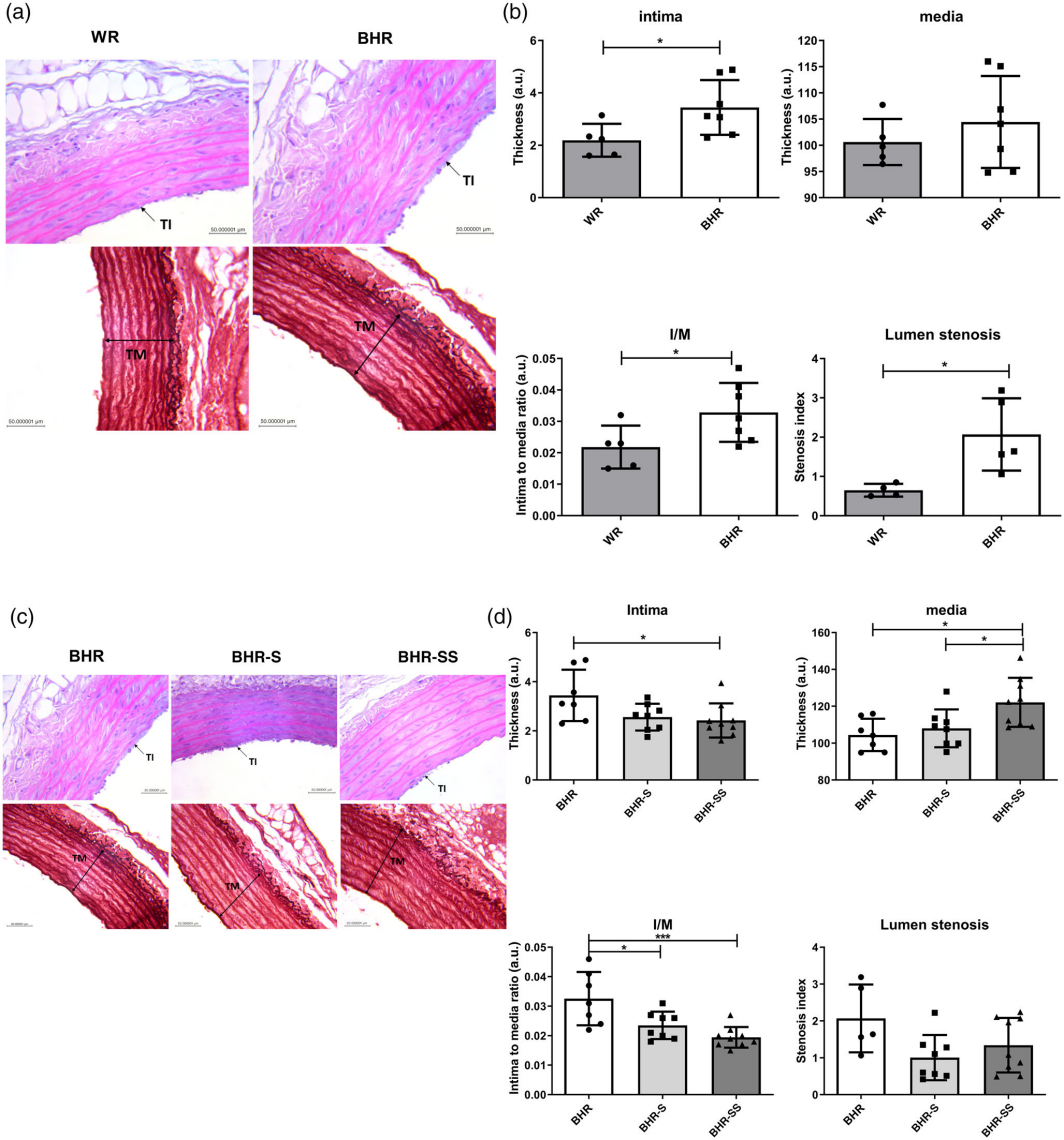
(a,b) Between‐strain differences in morphology of arteries [(a) original micrographs; (b) individual intima thickness, media thickness, intima‐to‐media ratio and lumen stenosis values, and mean values ± SD]. (c,d) Effect of salt and stress on morphology in BHR aorta [(c) original micrographs; (d) intima thickness, media thickness, intima‐to‐media ratio and lumen stenosis values as well as means ± SD]. Statistical significance was tested by Student's *t*‐test for independent samples by groups (b) or one‐way ANOVA, with post hoc comparison by Tukey's HSD test (d). Statistical significance: ^*^ *P* < 0.05 and ^***^ *P* < 0.001. Abbreviations: BHR, borderline hypertensive rat; BHR‐S, borderline hypertensive rat exposed to saline; BHR‐SS, borderline hypertensive rat exposed to saline and repeated heterotypic stress; I/M, intima‐to‐media ratio; WR, Wistar rat.

Strain comparison showed that the intima was thicker in BHRs compared with WRs (*P* = 0.03886; Figure 2a,b). The intima‐to‐media ratio (*P* = 0.04938) and the percentage of lumen stenosis were higher in BHRs (*P* = 0.01976). There was no difference in media thickness between BHRs and WRs (*P* = 0.39508).

Intima thickness was significantly higher in the BHR group compared with the BHR‐SS group (Figure 2c,d; *P* = 0.040834). There was no difference between BHR and BHR‐S groups (*P* = 0.09069) or between BHR‐S and BHR‐SS groups (*P* = 0.93479) in intima thickness. Media thickness was higher in the BHR‐SS group compared with both the BHR group (*P* = 0.013163) and the BHR‐S group (*P* = 0.04285). When exposed to elevated salt intake or to salt and stress, BHRs showed a small decrease in intima‐to‐media ratio (*P* = 0.019019 and *P* = 0.000844, respectively). Treatment with high salt intake with or without stress had no significant effect on the stenosis index (one‐way ANOVA, *P* = 0.063368).

### 3.3 Antioxidant enzymes activity and protein levels

#### 3.3.1 Aorta: WR versus BHR

**FIGURE 3:**
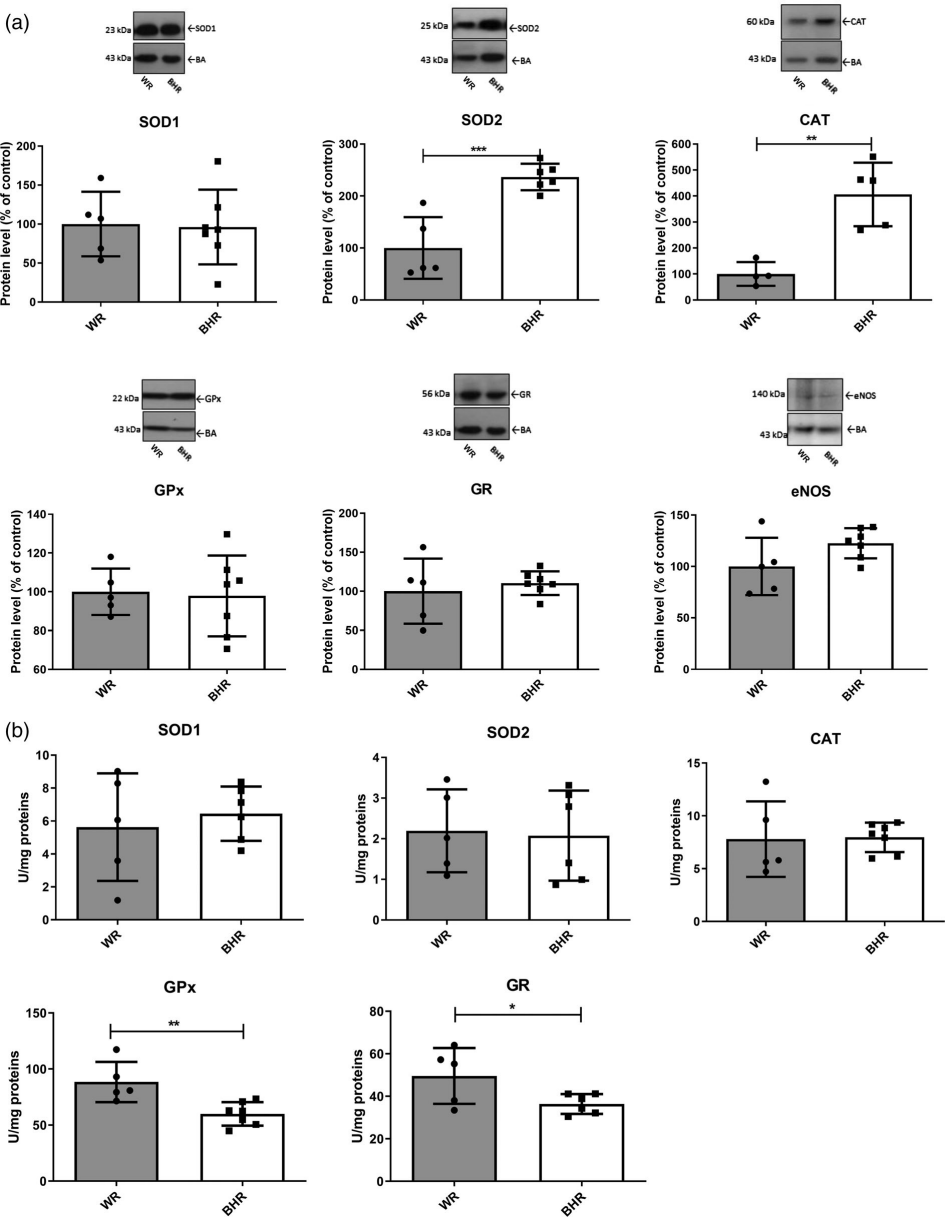
Antioxidant enzyme protein levels (a) and activities (b) of the aorta in WR and BHR animals. Individual values are presented, in addition to means ± SD. Statistical significance was tested by Student's *t*‐test for independent samples by groups. Statistical significance: ^*^ *P* < 0.05, ^**^ *P* < 0.01 and ^***^ *P* < 0.001. Abbreviations: BHR, borderline hypertensive rat; CAT, catalase; eNOS, endothelial nitric oxide synthase; GPx, glutathione peroxidase; GR, glutathione reductase; SOD1, copper–zinc superoxide dismutase; SOD2, manganese superoxide dismutase; WR, Wistar rat.

In comparison to normotensive WRs, BHRs kept in baseline conditions exhibited higher protein levels of SOD2 (*P* = 0.00061) and CAT (*P* = 0.002216), but lower activities of GPx (*P* = 0.00581) and GR (*P* = 0.04598) in the aorta (Figure 3a,b).

#### 3.3.2 Borderline hypertensive rat aorta: Effect of high salt intake and stress

**FIGURE 4:**
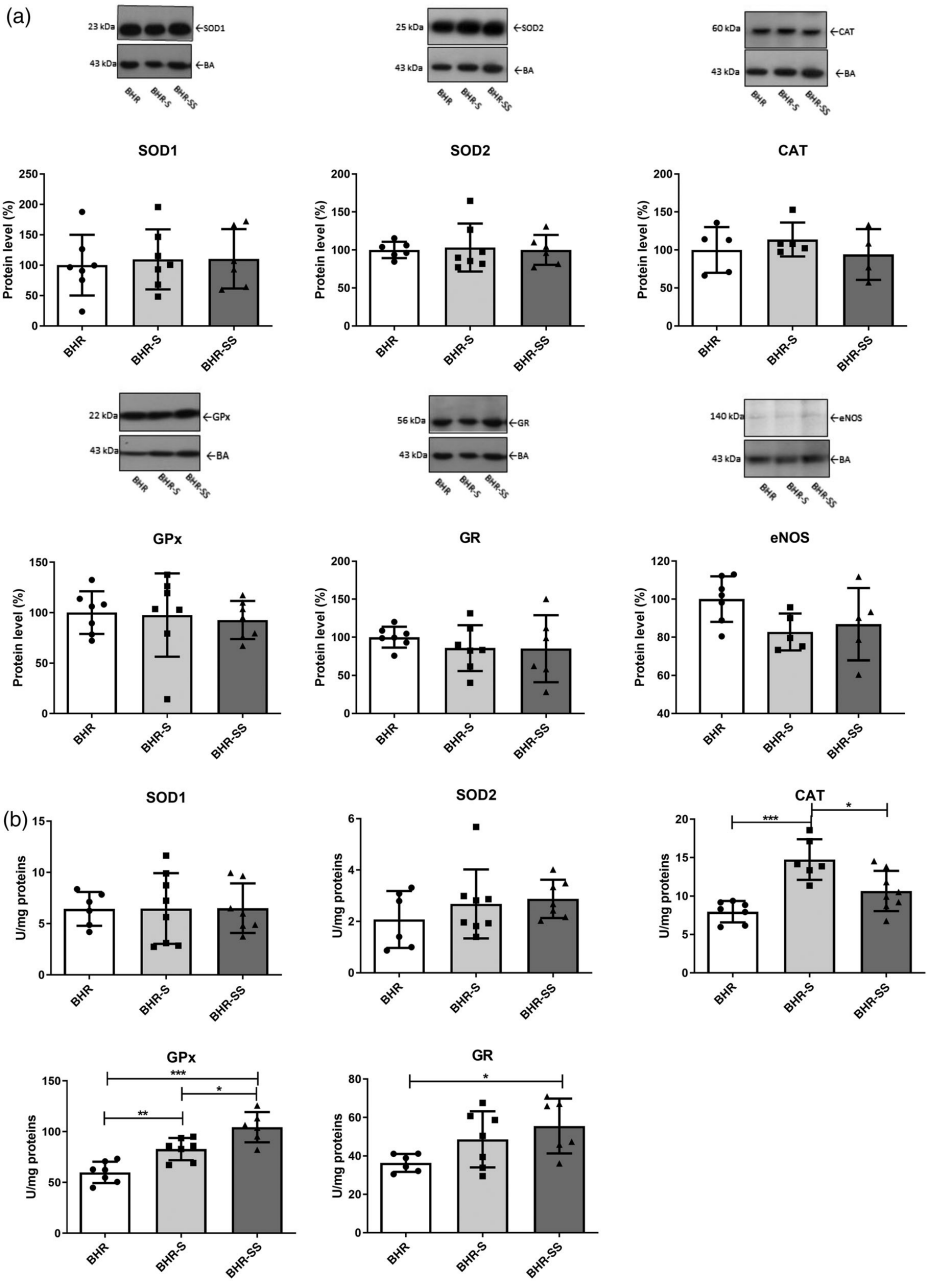
Antioxidant enzyme protein levels (a) and activities (b) of the aorta in BHR, BHR‐S and BHR‐SS groups. Individual values are presented, in addition to the means ± SD. Statistical significance was tested by one‐way ANOVA, with post hoc comparison by Tukey's HSD test. Statistical significance: ^*^ *P* < 0.05, ^**^ *P* < 0.01 and ^***^ *P* < 0.001. Abbreviations: BHR, borderline hypertensive rat; BHR‐S, borderline hypertensive rat exposed to saline; BHR‐SS, borderline hypertensive rat exposed to saline and repeated heterotypic stress; CAT, catalase; eNOS, endothelial nitric oxide synthase; GPx, glutathione peroxidase; GR, glutathione reductase; SOD1, copper–zinc superoxide dismutase; SOD2, manganese superoxide dismutase; WR, Wistar rat.

Salt alone or in combination with stress had no effect on antioxidant enzyme and eNOS protein levels in the aorta of BHRs (Figure 4a).

High salt intake increased the activity of CAT (Figure 4b; significant ANOVA treatment effect, *F* = 14, *P* = 0.000198; Tukey's HSD post hoc test, *P* = 0.000263) and GPx (significant ANOVA treatment effect, *F* = 22, *P* < 0.0001; Tukey's HSD post hoc test, *P* = 0.00665) in BHR aorta. The combination of high salt intake and stress increased GPx (significant ANOVA treatment effect, *F* = 22, *P* < 0.0001; Tukey's HSD post hoc test, *P* = 0.000167) and GR (significant ANOVA treatment effect, *F* = 3.8, *P* = 0.045517; Tukey's HSD post hoc test, *P* = 0.03911) activities compared with the aorta of the BHR in baseline conditions (Figure 4b). However, CAT activity in the aorta of the BHR‐SS group was lower than in the BHR‐S group (*P* = 0.01075).

#### 3.3.3 Heart: WR versus BHR

**FIGURE 5:**
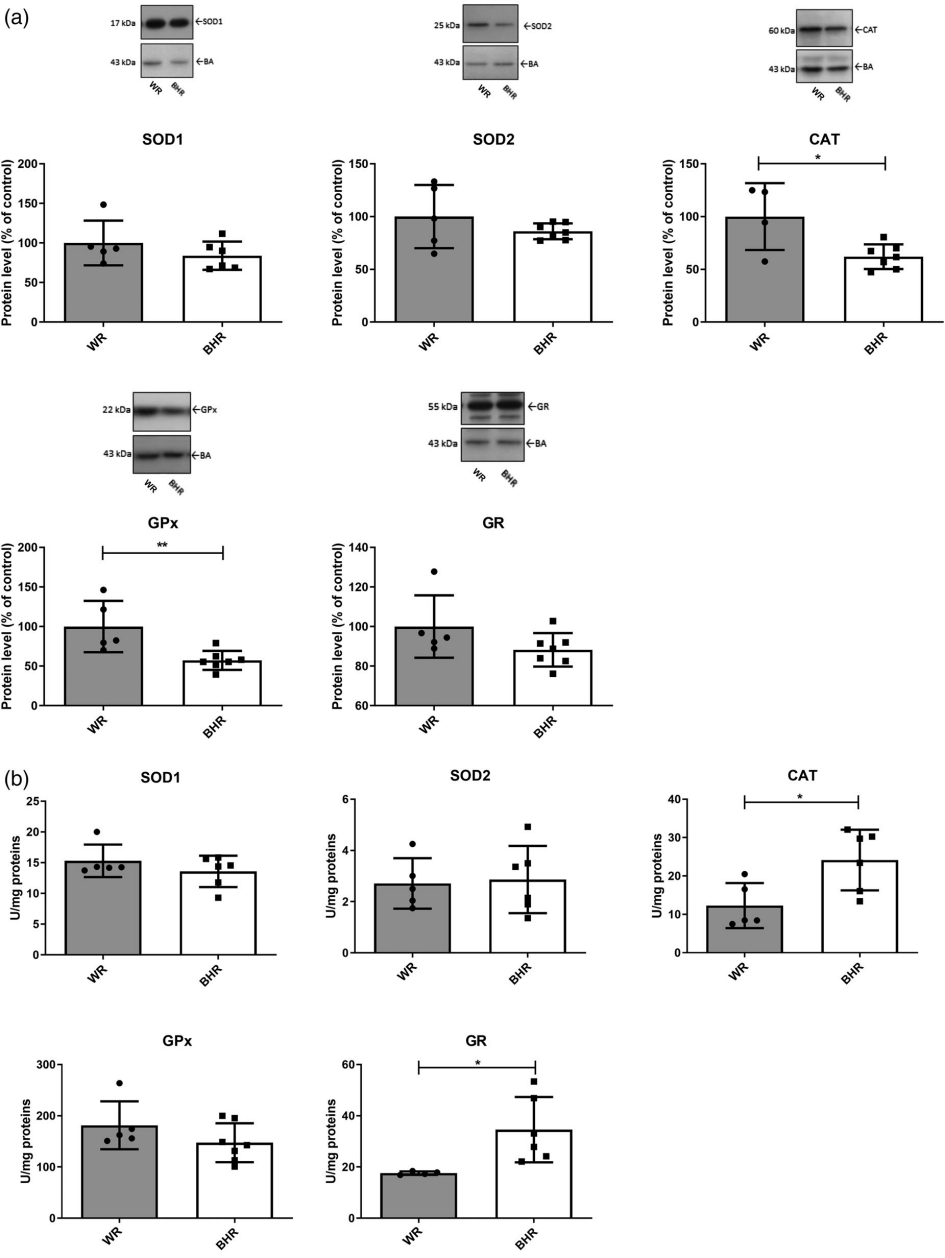
Antioxidant enzyme protein levels (a) and activities (b) in the heart of WR and BHR animals. Individual values are presented, in addition to means ± SD. Statistical significance was tested by Student's *t*‐test for independent samples by groups. Statistical significance: ^*^ *P* < 0.05 and ^**^ *P* < 0.01. Abbreviations: BHR, borderline hypertensive rat; CAT, catalase; GPx, glutathione peroxidase; GR, glutathione reductase; SOD1, copper–zinc superoxide dismutase; SOD2, manganese superoxide dismutase; WR, Wistar rat.

In baseline conditions, BHR hearts had reduced CAT (*P* = 0.016449) and GPx (*P* = 0.008892) protein levels compared with normotensive Wistar control animals (Figure 5a). However, the activities of CAT (*P* = 0.021675) and GR (*P* = 0.03159) in hearts of BHRs kept in baseline conditions were higher in comparison to normotensive Wistar control animals (Figure 5b).

#### 3.3.4 Borderline hypertensive rat heart: Effect of high salt intake and stress

**FIGURE 6:**
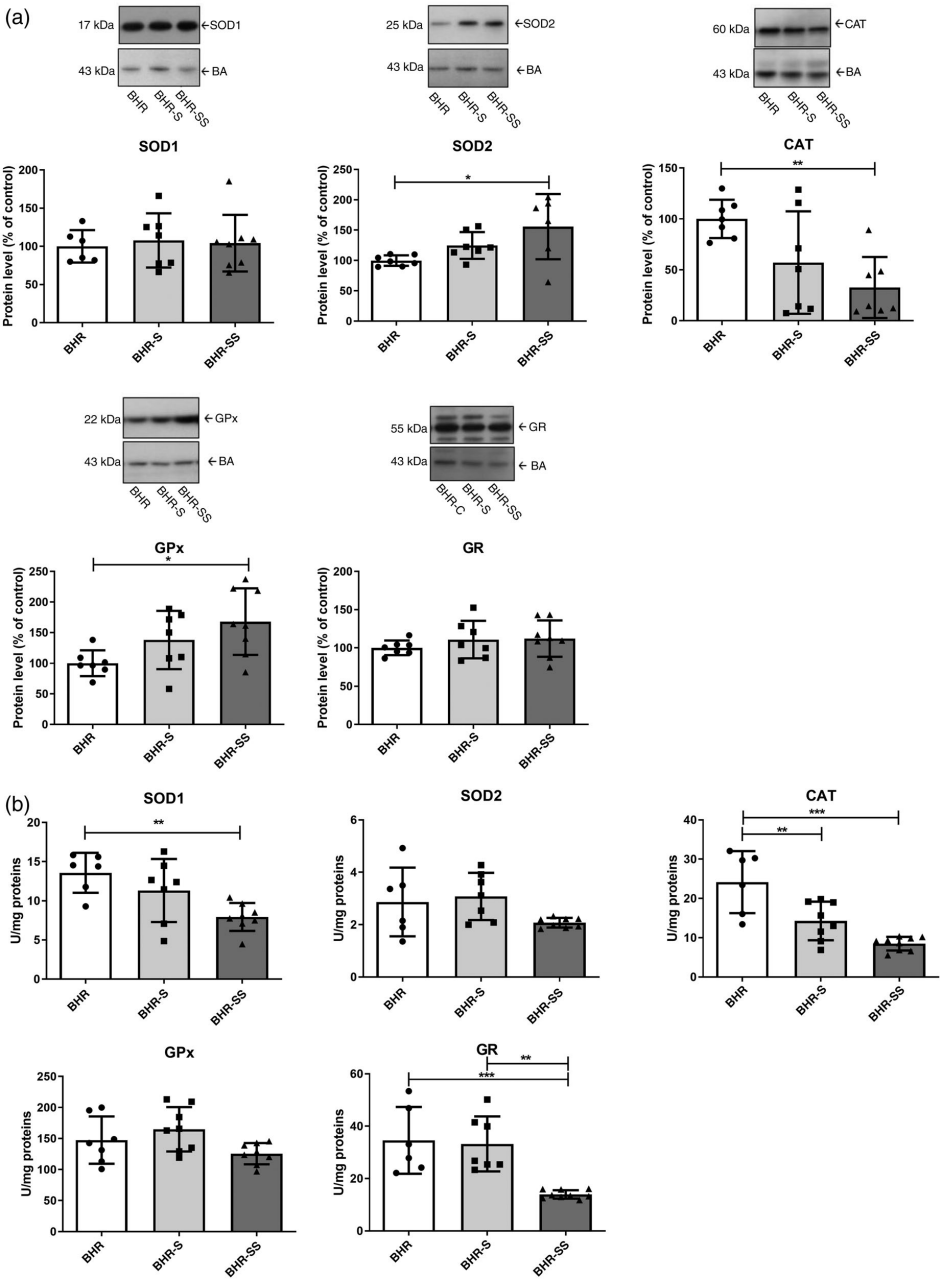
Antioxidant enzyme protein levels (a) and activities (b) in the heart of BHR, BHR‐S and BHR‐SS animals. Individual values are presented, in addition to means ± SD. Statistical significance was tested by one‐way ANOVA, with post hoc compared by Tukey's HSD test. Statistical significance: ^*^ *P* < 0.05, ^**^ *P* < 0.01 and ^***^ *P* < 0.001. Abbreviations: BHR, borderline hypertensive rat; BHR‐S, borderline hypertensive rat exposed to saline; BHR‐SS, borderline hypertensive rat exposed to saline and repeated heterotypic stress; CAT, catalase; GPx, glutathione peroxidase; GR, glutathione reductase; SOD1, copper–zinc superoxide dismutase; SOD2, manganese superoxide dismutase; WR, Wistar rat.

High salt intake had no effect on the protein levels of antioxidant enzymes in the heart, but the combination of high salt intake and stress decreased the protein levels of CAT (significant ANOVA, *F* = 6.5, *P* = 0.007698; Tukey's HSD post hoc test, *P* = 0.006274) and increased those of SOD2 (significant ANOVA, *F* = 3.4, *P* = 0.021936; Tukey's HSD post hoc test, *P* = 0.021303) and GPx (significant ANOVA, *F* = 4.4, *P* = 0.026408; Tukey's HSD post hoc test, *P* = 0.020416), in contrast to BHRs in baseline conditions (Figure 6a).

Elevated salt intake led to a decrease in CAT activity compared with the BHR group (significant ANOVA treatment effect, *F* = 17, *P* < 0.0001; Tukey's HSD post hoc test, *P* = 0.007714). A decrease in the activities of SOD1 (significant ANOVA treatment effect, *F* = 6.7, *P* = 0.006712; Tukey's HSD post hoc test, *P* = 0.005766), CAT (significant ANOVA treatment effect, *F* = 17, *P* < 0.0001; Tukey's HSD post hoc test, *P* = 0.00021) and GR (significant ANOVA treatment effect, *F* = 13, *P* = 0.0002319; Tukey's HSD post hoc test, *P* = 0.002081) were detected in hearts of the BHR‐SS group compared with the BHR group (Figure 6b). Lower GR activity was also confirmed in the BHR‐SS group compared with the BHR‐S group (significant ANOVA treatment effect, *F* = 13, *P* = 0.0002319; *P* = 0.001931).

## 4 DISCUSSION

Our results show that BHRs, although genetically predisposed to hypertension, develop overtly high BP after exposure to additional risk factors, such as increased salt intake and stress, that accelerate the transition of prehypertension to hypertension. At the age of 36 weeks, antioxidant levels in BHRs are dissimilar regarding both the activity and protein content compared with age‐matched WRs, suggesting a different oxidative state. Moreover, exposure of BHRs to high salt intake and stress led to further changes in individual antioxidant components that suggest specific tissue ROS production. The process develops slowly, given that no difference in antioxidant levels of juvenile BHRs was reported, and it seems to be a consequence rather than the cause of hypertension (Horvathova et al., 2016).

There was a higher basal level of SBP, DBP and MAP in BHRs compared with WRs, but exposure of BHRs to increased salt intake led to an additional increase in SBP at the 24th week, introducing these animals into a state of overt hypertension. It seems that in BHRs, an elevated level of circulating plasma noradrenaline changes vascular reactivity and contributes to differential haemodynamic adaptations (Fuchs et al., 1998). The BHRs are characterized by a high plasma concentration of vasopressin (Savic et al., 2020), and exogenous vasopressin provoked pressor‐induced cardiac hypertrophy (Fuchs et al., 1998). Furthermore, crowding stress elevates plasma corticosterone and accelerates development of hypertension in juvenile male (Bernatova et al., 2018) and female BHRs (Slezak et al., 2014). In our experiments, hypertension induced by high salt intake and stress led to a decrease of intima thickness and an increase of media thickness, which reduced the intima‐to‐media ratio in BHRs, suggesting an adaptive response to elevated BP.

To gain a better understanding of basic oxidative processes and the role of antioxidant enzymes in the development of hypertension in BHRs, we examined changes of antioxidative enzyme protein levels and activity in BHRs kept in baseline conditions compared with WRs and with BHRs exposed to high salt with or without stress. Our results suggest that ROS production and/or elimination in WRs and BHRs varies, given that differences in basal antioxidant enzymes levels were noted. Exposure to salt and stress led to adaptive response in BHR aorta and heart in a tissue‐specific manner.

In the aorta of the BHRs, protein levels of SOD2 and CAT were higher, suggesting elevated constitutive ROS formation and elimination, compared with normotensive WRs. It has been shown that overexpression of SOD2 in pulmonary artery smooth muscle cells increases the Ca^2+^ response but has no effect on the redox indicator (Waypa et al., 2006), suggesting a change in the ROS‐mediated physiological response and reactivity. The elevated SOD2 protein level observed in our experiment also suggests the importance of mitochondrial superoxide elimination and/or H_2_O_2_ production. On the one hand, it is important to prevent reaction of superoxide with NO that can lead to impairment of NO signalling and/or enhanced production of peroxynitrite that causes oxidative stress. On the other hand, elevated H_2_O_2_ can enhance sensitivity to Ca^2+^ signalling and thus accelerate physiological reactions. Additionally, the level of CAT protein is elevated in the BHR aorta, suggesting that this tissue is prepared for H_2_O_2_ removal and tuning down the effects of H_2_O_2_. Taken together, these processes imply that antioxidant enzyme action is directed towards higher or preserved NO reactivity. There are many studies suggesting that overall NO reactivity is damaged in high‐salt conditions (Campese et al., 1996; Fujiwara et al., 2000). Also, there is evidence that stress alters the mechanism of endothelium‐dependent relaxation (Fuchs et al., 1998). However, our western blot analysis could not show the inducible nitric oxide synthase protein level in aorta (data not shown), whereas the eNOS protein level did not display intergroup variation. Altogether, these results indicate significant importance of antioxidant enzyme activity in preserving the NO‐mediated signalling pathway, given that we did not detect modulation of NO synthase activity. In young BHRs, despite elevated BP, no signs of oxidative damage of plasma lipids, NO deficiency or endothelial disfunction were observed in control BHRs vs. age‐matched Wistar Kyoto rats (Bernatova et al., 2018).

Although the protein levels of SOD2 and CAT in BHR aorta were higher, the enzymatic activity did not differ from that of WRs, suggesting post‐translational suppression of their activity. On the contrary, GPx and GR activity in BHRs was decreased in comparison to normotensive rats, suggesting a state of constitutive post‐translational inhibition of their activity. Overall, taken together, these data show that the increased protein level of SOD2/CAT is enough to establish a stable low ROS cellular state, especially impacting superoxide and H_2_O_2_ in the aorta of BHRs. Consequently, there is no need for high GPx and GR activity that would act in a co‐ordinated manner for H_2_O_2_ elimination.

In the aorta, our experimental treatments (high salt intake and stress) affected only enzyme activity, whereas protein levels remained unchanged, which implicates a response to transient modulation of redox status. High salt intake provoked an increase in CAT and GPx activities in the aorta, suggesting elevation of both H_2_O_2_ production and its elimination. Again, this emphasizes the importance of H_2_O_2_ removal in the BHR aorta because of its influence on Ca^2+^ reactivity. Eradication of H_2_O_2_ in BHR aorta seems to be of the utmost importance; BHR aorta had higher protein levels of CAT, and its activity was additionally elevated after high salt intake. Stress provoked an increase in both GPx and GR activities, suggesting that the elimination of H_2_O_2_ is imperative. These elevations appear to be at the level of post‐translational regulation and could involve different modalities, ranging from regulation of activity by substrates and/or products to phosphorylation and methylation. It seems that intake of salt leads to increased blood volume, loads blood vessels and increases the pressure of H_2_O_2_, which is accompanied by an increase in CAT activity in the aorta. Exposure to stress decreases this effect of salt, probably owing to the secretion of adrenaline, which increases HR and blood flow. Furthermore, repeated stress diminishes the peroxide pressure in the system and heightens the activity of GPx. Although effects of chronic stress depend on developmental age, it seems that key regulators of stress effects on vascular reactivity are superoxide anions, vasoconstrictor cyclooxygenase products and a loss of K^+^ channel‐mediated relaxation (Giulumian et al., 1999). In our experiment, the elevation of GPx and GR found in BHR thoracic aorta after exposure to stress shows promoted protection against lipid peroxidation and thus, an increase of cyclooxygenase products. It is known that nitrovasodilators relax mesenteric microvessels by cGMP‐induced stimulation of Ca^2+^‐activated K^+^ channels (Carrier et al., 1997), suggesting the importance of preserving NO‐dependant Ca^2+^ signalling. Therefore, an increase of GPx and GR also has a role in preventing the influence of H_2_O_2_ on Ca^2+^ channels, to maintain the NO‐mediated vasodilatory response physiologically. Crowding stress did not reduce NO production in the aorta or acetylcholine‐induced relaxation of the femoral arteries in BHRs, but reduced aortic NO production was observed 2 weeks post‐stress, suggesting a reduction of NO‐dependent vasorelaxation components (Bernatova et al., 2018).

In the heart, lower levels of CAT and GPx proteins in BHRs are suggestive of lower constitutive H_2_O_2_ production or higher H_2_O_2_ tolerance compared with normotensive WR counterparts. Hydrogen peroxide is a known redox modulator of various types of Ca^2+^ channels (Bogeski et al., 2011), and the difference in protein levels of CAT and GPx implies dissimilarity in basal ROS homeostasis of BHRs and WRs. That is achieved by alternative redox homeostatic regulation of heart functionality. Coordinated expression and/or downregulation of peroxisomal CAT and cytoplasmic GPx‐1 seems to be achieved by FoxO (family of Forkhead transcription factors)‐regulated elements, suggesting that H_2_O_2_ might be reduced in multiple cellular compartments in parallel (Klotz et al., 2015). However, given that CAT activity is elevated, it seems that ROS homeostasis in the BHR heart is achieved by post‐translational elevation of CAT activity, in addition to GR, indicating faster elimination of H_2_O_2_ and higher GSH turnover. Activity of CAT is known also to be regulated by the tyrosine‐protein kinase c‐Abl/Arg pathway. In conditions of low H_2_O_2_ concentration, phosphorylation of CAT is promoted, leading to its enhanced activity, but as the H_2_O_2_ level increases further, c‐Abl/Arg dissociates from CAT, induces dephosphorylation and lowers activity (Cao et al., 2003). Therefore, our results suggest higher decomposition of H_2_O_2_ in the BHR heart, but at relatively low H_2_O_2_ levels that do not provoke higher protein levels of CAT or GR. Furthermore, it is implied that, in BHR heart, sensitivity of Ca^2+^ channels and/or the Ca^2+^ response to H_2_O_2_ is kept low compared with WRs. It is known that H_2_O_2_ is an important factor contributing to the generation of rhythm disturbances and cellular damage during reperfusion of previously ischaemic myocardium. Also, direct application of H_2_O_2_ initiates proarrhythmic activity (Zhao et al., 2020). In our experiment, we found differences in HR that might reflect H_2_O_2_‐mediated heart functionality. H_2_O_2_ can increase ATP‐sensitive K^+^ current (Zhao et al., 2020) and inward‐rectifying K^+^ current (Bychkov et al., 1999), which can decrease the delayed rectifier K^+^ current (Bae et al., 2016). H_2_O_2_ appears to have a number of different effects on L‐type Ca^2+^ currents in rat ventricular myocytes, from slowing the rate of fast inactivation to a biphasic effect on the spontaneous cycle length (Bao et al., 2000). We suggest that the maintenance of ROS homeostasis in the heart of the BHR and normotensive WR is achieved in different ways, affecting a particular ROS (i.e. H_2_O_2_) production/elimination rate that regulates heart function specifically.

The effect of salt depends on daily intake, intake frequency, duration of treatment and developmental stage (Rust & Ekmekcioglu, 2017). In our experiment, high salt intake did not provoke any response at the level of antioxidant enzymes in the BHR heart, suggesting high salt tolerance. The only enzyme in BHR heart affected by high salt intake was CAT, defined by lower activity, probably owing to decreased demand for H_2_O_2_ removal. However, when combined with salt, stress caused significant changes and perturbations to redox and ROS homeostasis in BHR heart. Repeated heterotypic stress led to an additional reduction in CAT activity and in SOD1 and GR activities. It also led to a decline of CAT and increase of SOD2 and GPx protein levels, suggesting lowered protection against H_2_O_2_, but elevated defence towards lipid peroxides. Diminished CAT activity can be a consequence of declining cytosolic SOD1 activity. Given that there are no changes in protein levels and minor changes in the activity of antioxidant enzymes present in the heart after high salt intake, our results show that stress is a major factor for ROS and redox disbalance parallel to establishment of high BP in BHRs prone to hypertension.

Overall, high salt intake and repeated heterotypic stress raised BP in the BHRs. The results suggest that the BHR heart is fundamentally less exposed than aorta, and accommodates sufficiently to lower H_2_O_2_ levels. In contrast, in the BHR aorta, constitutive exposure to ROS (superoxide and H_2_O_2_) seems to be higher, but protein levels of SOD2 and CAT, in addition to post‐translational modification of glutathione‐dependent enzymes, are efficiently tuned towards optimal protection and preservation of reductive cellular cofactors. Both results suggest that in the BHR, antioxidant enzymes, apart from their apparent antioxidant role, also subtly regulate the ROS signalling function. In line with the observed differences in basal levels of antioxidant enzymes in the heart and aorta of normotensive WRs and BHRs, our results point out a significant role of redox‐dependent processes in the development of hypertension. Exposure of BHRs to salt and stress led to elevation of antioxidative defence in the aorta, but to its decrease in the heart. This suggests that BHR hearts are adapted to oxidative pressure, compared with the aorta, which seems particularly sensitive. Sensitivity of the aorta could be, in part, a consequence of impaired redox processes that influence a cascade of signalling events and provoke additional oxidative pressure. Further studies, in line with these results, could shed more light on the exact role of ROS in the development of hypertension in borderline hypertensive individuals.

## AUTHOR CONTRIBUTIONS

Conception or design of the work: Bojana Savić, Olivera Šarenac, Zorana Oreščanin Dušić, David Murphy, Nina Japundžić‐Žigon and Sofija Glumac. Acquisition or analysis or interpretation of data for the work: Bojana Savić, Olivera Šarenac, Jelena Brkljačić, Zorana Oreščanin Dušić, Sofija Glumac and Duško Blagojević. Drafting the work or revising it critically for important intellectual content: Zorana Oreščanin Dušić, Jelena Brkljačić, Bojana Savić, Duško Blagojević, David Murphy and Nina Japundžić‐Žigon. All authors agree to be accountable for all aspects of the work in ensuring that questions related to the accuracy or integrity of any part of the work are appropriately investigated and resolved. All persons designated as authors qualify for authorship, and all those who qualify for authorship are listed.

## CONFLICT OF INTEREST

The authors declare no conflict of interest.

## Supporting information


Statistical Summary Document

## Data Availability

The data that support the findings of this study are available from the corresponding author, upon reasonable request.
